# Sex-related differences in gene expression in early-stage bladder cancer revealed by whole-transcriptome sequencing

**DOI:** 10.1186/s12885-026-15666-3

**Published:** 2026-02-09

**Authors:** Natalia Zeber-Lubecka, Konrad Bilski, Michalina Dąbrowska, Krzysztof Goryca, Joanna Ziemska-Legięcka, Jerzy Ostrowski, Jakub Dobruch, Ewa E. Hennig

**Affiliations:** 1 Department of Gastroenterology, Hepatology and Clinical Oncology, Centre of Postgraduate Medical Education, 02-781 Warsaw, Poland; 2 Department of Genetics, Maria Sklodowska-Curie National Research Institute of Oncology, 02-781 Warsaw, Poland; 3 Department of Urology, Centre of Postgraduate Medical Education, Independent Public Hospital of Prof. W. Orlowski, 00-416 Warsaw, Poland

**Keywords:** Non, Muscle, Invasive bladder cancer; early, Stage cancer; sex, Related; low, Grade stage; differentially expressed genes

## Abstract

**Background:**

Bladder cancer (BC) is significantly more prevalent in men than in women, yet female patients often experience higher recurrence rates and poorer prognosis.

**Methods:**

Our study aimed to investigate sex-specific gene expression differences in early-stage BC using whole-transcriptome sequencing. A total of 51 patients diagnosed with low-grade Ta stage non-muscle-invasive bladder cancer were recruited. Paired tissue samples from tumor lesions and adjacent healthy bladder mucosa (BM) were analyzed to identify differentially expressed genes (DEGs).

**Results:**

Among the top 100 most significant DEGs for each gender, overwhelmingly more upregulated in BC comparing with BM were in male than female tissues (90% vs. 19%). The most significantly altered expression in female BC tissues included *MT-ND6, ARL4C, ASGR1, MYBL1*, and *SCAMP5*, whereas in males, *ONECUT2, SPEG, CTSE, GJB2*, and *SYNM*. Notably, 753 DEGs were unique to female patients, while 3989 were specific to males, with 1633 shared between both sexes. Functional annotation revealed that female-unique DEGs were significantly enriched in immune-related pathways, including regulation of leukocyte activation and cell–cell adhesion, and lymphocyte differentiation. Whereas male-unique DEGs were predominantly associated with pathways related to cell cycle regulation, mitochondrial function, and androgen receptor signaling. Immune-related gene expression indicated that female-specific DEGs were involved in leukocyte activation and antigen receptor signaling, whereas male-specific DEGs were linked to B-cell activation and neutrophil-mediated immune responses. A two-factor interaction model identified *S100A14* as the only protein-coding gene whose expression exhibited a significant sex-dependent pattern, with four additional genes (*GJB2, DSC2, TM4SF* and *ALOX15B*) showing a probable interaction effect.

**Conclusions:**

These findings provide preliminary evidence supporting further investigation of sex-specific approaches to BC management.

**Supplementary Information:**

The online version contains supplementary material available at 10.1186/s12885-026-15666-3.

## Introduction

Bladder cancer (BC) occurs 3–4 times more frequently in men than in women [1], which is attributed to differences in exposure to risk factors such as smoking and occupational chemical exposure. However, there is evidence suggesting that female gender may be an indicator of higher risk of BC recurrence, a poorer prognosis, and higher cancer-specific mortality, which could be related to differences in tumor biology, including gene expression [2].

Non-muscle-invasive bladder cancers (NMIBC), previously referred to as superficial BC, are malignant tumors of the urothelium that have not penetrated the detrusor muscle. Around 70% of NMIBC cases are low-grade classified as non-invasive to muscle, developed as superficial papillary outgrowths at the time of diagnosis [3]. Among these, approximately 70% are stage Ta, 20% are T1, and 10% are carcinoma in situ [4]. These lesions are often associated with *FGFR3* mutations and carry a high risk of recurrence. However, they typically exhibit a low likelihood of progressing to high-grade lesions, invading deeper tissues, or metastasizing [3]. Low-grade Ta stage lesions, which means that tumor is located only in the innermost layer of the bladder lining, have a recurrence rate between 50 and 70%, with progression occurring in around 5% of cases [5]. In contrast, high-grade T1 lesions recur in over 80% of instances and progress in 50% of patients within three years [6]. Standard treatment for NMIBC includes transurethral resection of the bladder tumor (TURBT), often followed by adjuvant intravesical chemotherapy or Bacillus Calmette-Guérin therapy.

Recent findings suggest that, beyond well-known sex-biased factors, more subtle influences, such as genetic imbalances and epigenetic mechanisms, have been overlooked due to their complexity and experimental challenges [2]. One of the key factor of sex-specific differences in BC is the role of sex hormones and their receptors. Studies suggest that androgens and their receptors (ARs) may promote tumor growth in males, while estrogen and estrogen receptors might have a protective effect in females [7, 8]. This hormonal influence contributes to distinct gene expression profiles in both sexes [9]. Men often exhibit higher expression of genes related to tumor proliferation, while women may show differences in genes involved in immune response and inflammation [10]. Elevated estrogen levels in women may drive differences in immune response by upregulating immune-related genes, including those involved in IFN-ɣ expression [11]. This enhanced immune response may contribute to the lower incidence of BC in females, potentially offering anti-tumor immunity. In turn, higher testosterone levels in men may promote increased Th1 cytokine production, potentially leading to a lower incidence of adverse BC disease stage compared to women [10].

Additionally, the X chromosome, which contains genes that can escape inactivation in females, may provide a protective effect, adding another layer of complexity to understanding these sex-specific gene expression patterns. To maintain gene dosage, epigenetic modifications randomly inactivate one X chromosome, though about 15% of genes, including immunity-related ones like *TLR7*, escape inactivation. Higher *TLR7* expression on B cells in females may lead to increased IgG production, contributing to sex-associated immune responses in cancer. While *CXCL13* is a promising biomarker for favorable outcomes, its high expression in female patients with NMIBC is linked to high-grade tumors and increased B cell recruitment [12]. Inactivation or decreased expression of *KDM6A,* another gene which escapes X-chromosome inactivation, has been linked to poorer outcomes, especially in women, highlighting its importance in the disease's progression and prognosis [13]. Also, mutations in *KDM6A*, which are associated with reduced infiltration of immune cells within the tumor, increased inflammatory signaling, and enhanced immune evasion [14], are more commonly observed in female BC patients, suggesting a gender-specific vulnerability. On the other hand, male patients with BC exhibit a higher frequency of *TP53* mutations compared to female patients [15]. All these factors, along with higher PD-L1 levels and distinct immune gene expression, may contribute to differences in BC between genders, influencing resistance to immunotherapy and shorter progression-free survival in females [12].

Although significant preclinical advancements have been made in the field, male and female BC patients continue to receive very similar clinical treatment, apart from differences related to anatomy [2]. The high rates of recurrence, resistance to conventional therapies, and the economic strain associated with BC highlight the need for a long-overdue shift towards precision medicine that accounts for biological sex. This approach could enhance sex-specific survival outcomes and reduce treatment costs. Consistently, the aim of this study was to determine the gender-related differences in gene expression using whole transcriptome sequencing (WTS) in early-stage BC. Paired tissue samples of low-grade Ta stage BC and adjacent healthy-appearing bladder mucosa (BM) were collected from men and women. We have selected low-grade Ta tumors as they represent the most frequent NMIBC subtype and provide a clinically relevant model for studying early sex-related molecular differences without confounding effects of advanced disease. WTS based analysis revealed substantial sex-specific differences in gene expression profiles between BC and adjacent BM tissues, emphasizing the need to integrate biological sex into precision medicine strategies for BC treatment.

## Materials and methods

### Participants and samples collection

The study was approved by the local ethics committee (Centre of Postgraduate Medical Education, Warsaw, Poland, project ID: 125/PB/2018), and the study protocol conforms to the ethical guidelines of the 1975 Declaration of Helsinki. All participants provided informed written consent prior to the examination.

In total, 52 patients (22 female and 30 male) with low-grade Ta stage BC tumors were recruited to the study. The inclusion criteria were as follows: (1) age over 18 years; (2) no bladder catheter in place; and (3) absence of symptoms or signs of an active urinary tract infection. The exclusion criteria were: (1) inability to meet the inclusion criteria; (2) refusal to provide informed consent; (3) the presence of muscle-invasive BC; and (4) the presence of NMIBC other than low-grade Ta stage.

BC tumor and adjacent healthy-appearing BM tissues were collected during TURBT procedure. BM specimens were collected from regions that appeared normal under endoscopic inspection, with no suspicious or abnormal changes (i.e., no erythema, edema, thickening, papillary or nodular appearance). Healthy-appearing mucosa was defined as tissue with a smooth surface, normal vascular pattern, and no endoscopic features suggestive of carcinoma in situ or inflammation. It was sampled from a region distant from the tumor margin (typically on the opposite bladder wall or at least several centimeters away) to reduce potential microenvironmental influence. Adjunct imaging modalities were not required in this clinical setting, as standard white-light cystoscopy is sufficient for papillary low-grade Ta lesions in routine practice (EAU guidelines, Sects. 5.8 & 5.10) [16]. These criteria ensured that the control mucosa was not directly affected by the tumor, while remaining within the same bladder environment. Half of the collected BC and BM specimens were snap-frozen and stored at −80 °C until processing, and the other half was used for histopathological evaluation.

### Sample processing and whole transcriptome sequencing

Total RNA that was isolated from fresh frozen BC tumor and BM tissues using the mirVana™ PARIS™ RNA and Native Protein Purification Kit (Thermo Fisher Scientific, Waltham, MA, USA), following the manufacturer's instructions. Isolated RNA concentration and its purity were assessed by NanoDrop™ 2000 spectrophotometer (Thermo Fisher Scientific, Waltham, MA, USA) determining the A260/280 and A260/230 ratios, respectively. RNA integrity number (RIN) and distribution value 200 (DV200) were assessed with an Agilent RNA 6000 Nano Kit on a 2100 Bioanalyzer (Agilent, Santa Clara, CA, USA). The RNA isolates were stored at −80° C. Next, 10 ng of RNA was used to prepare the RNA libraries. During the library preparation, rRNA was depleted using KAPA RNA HyperPrep with RiboErase and KAPA Globin Depletion Hybridization Oligos (Roche, Basel, Switzerland). SMART-Seq Stranded Kit (Takara Bio, Kusatsu, Japan) was used for low input RNA quantities. The libraries were sequenced on the NovaSeq 6000 platform (Illumina, San Diego, CA, USA). Depths of 30 million (6 Gb) paired-end 100 bp reads were generated for each sample.

### Sequencing data and statistical analyses

Raw sequences were trimmed according to quality using Trimmomatic (version 0.39) with the following parameters: SLIDINGWINDOW:4:20 MINLEN:20. Trimmed sequences were mapped to human reference genome provided by Ensembl (version grch38_snp_tran) using Hisat2 with default parameters. Optical duplicates were removed using MarkDuplicates tool from Genome Analysis Toolkit package (version 4.2.3.0) with default parameters except optical_duplicate_pixel_distance set to 12,000. Mapped reads were associated with transcripts from grch38 database (Ensembl, version 109) using HTSeq-count (version 2.0.1) with default parameters except stranded set to “reverse”. Differentially expressed genes were selected using DESeq2 package (version 1.42.1). Only genes with at least 1024 reads in total in at least one compared group were tested. A two-factor (sex*tissue) model was used to identify gender-related tumor gene expression changes. Men and BM were the baseline. Effect size confidence intervals were estimated with bootstrap method using 200 iterations. *P*-values were adjusted (*p*_adj_) for multiple testing using the Benjamini–Hochberg algorithm. *P*_adj_-values less than 0.05 were considered significant. Plots were made in R software using ggplot2 library.

To assess potential batch effects, principal component analysis (PCA) and hierarchical clustering were performed. No clustering by batch or sample collection parameters was observed, indicating minimal technical bias. Therefore, batch effects were not included as covariates in the differential expression analysis. The proportion of mitochondrial reads per sample was assessed using ANOVA (Supplementary Figure S1). No significant effects were observed (*p-*values 0.92, 0.63 and 0.083 for sex, tissue type and interaction sex*tissue, respectively), indicating that mitochondrial read abundance is not systematically biased across groups.

Overrepresentation of Biological Processes (BP) and Molecular Function (MF) term categories from the Gene Ontology (GO) database among the differentially expressed genes was assessed with the Cytoscape platform (version 3.6.1) in combination with the ClueGO plugin (version 2.5.1). Default settings were applied with *p*-values adjusted with the Benjamini–Hochberg correction and a significance threshold set at 0.05.

## Results

### Patients demographic and clinical characteristics

Tissue samples were collected from 144 consecutive patients who provided informed consent and underwent TURBT. After pathological and molecular examinations, the data of all patients were further scrutinized and only those with non-invasive (Ta) and low-grade disease were included into final analysis. Table 1 presents their characteristics with respect to sexes and tumor features. Twenty two of them were female (42.3%). Mean age was 73 years for both sexes, ranging 47–84 years for women and 60–95 for men. None of the characteristics show statistically significant differences between males and females (*p* < 0.05).

**Table 1:** Patients demographic and clinical characteristics

	**Female** *N* = 22 (42.3%)	**Male** *N* = 30 (57.7%)	***p*-value**
**Demographics**			
Mean age; years [range] Current or former smoker	73 [47–84] 15 (68.2)	73 [60–95] 16 (53.3)	1.00 0.392
**Tumor characteristics**			
Single tumor	18 (81.8)	22 (73.3)	0.526
Tumor size > 3 cm	5 (22.7)	8 (26.7)	1.00
Tumor size < 3 cm	17 (77.3)	22 (73.3)	
Primary tumor	11 (50.0)	9 (30)	0.162
Recurrent tumor	11 (50.0)	21 (70)	

*P*-value estimated by chi-squared and Mann–Whitney *U*-test

### Identification of differentially expressed genes by whole transcriptome sequencing

**Fig. 1:**
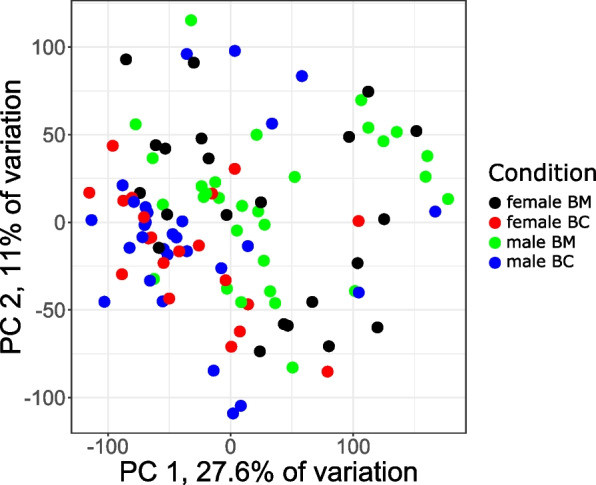
Principal component analysis (PCA) based on transcriptome profiling of samples sorted by gender and tissue origin. BC, bladder cancer; BM, healthy-appearing bladder mucosa

WTS of total RNA samples was used to establish gene expression profiles in low-grade Ta stage BC tumors and adjacent healthy-appearing BM tissues. Sequencing results for 49 BC and 52 BM samples of both sexes passed quality control and were further analyzed. Noticeable expression (i.e. with a base mean for genes with at least 1024 reads in total in at least one compared group) was shown for 16,324 genes in female samples and 18,925 genes in male samples. PCA based on gene expression profiles revealed significant clustering of samples by tissue origin (BC vs. BM), with PC1 *p*_adj_-value of 0.0025 for male and 0.032 for female samples, but no significant clustering by gender (Fig. 1).

Independently for women and men, pairwise comparisons were performed between BC and BM tissue samples identifying 2391 and 5640 protein-coding, differentially expressed genes (DEGs) (*p*_*adj*_ ≤ 0.05), presented in Supplementary Tables S1 and S2, respectively. Of these, 781 (32.7%) and 2971 (52.7%) protein-coding genes were more highly expressed in female and male BC samples, respectively. Among the top 100 most significant DEGs for each gender, 19% were upregulated in female tumor tissues and 90% in male tumor tissues.

**Fig. 2:**
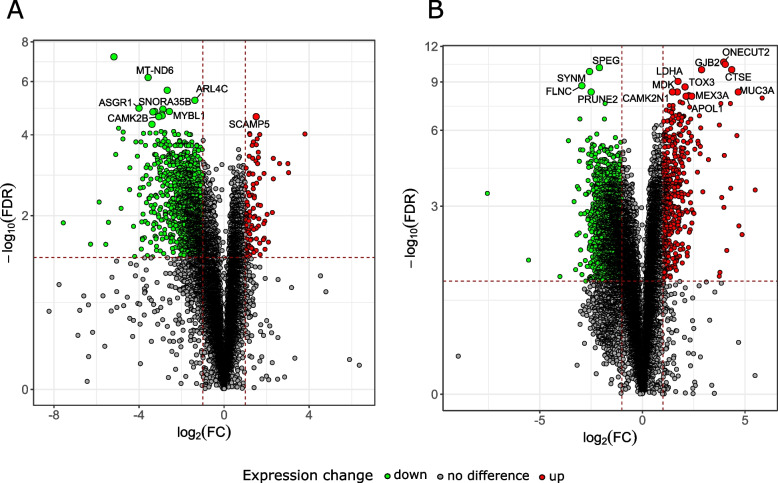
Volcano plots showing significant differentially expressed genes (DEGs). The red and green dots indicate genes with increased expression (Up) and decreased expression (Down), respectively. The top significant DEGs between bladder cancer (BC) and healthy-appearing bladder mucosa (BM) tissues were indicated in each plot; (**A**) female samples; (**B**) male samples

Volcano plots, commonly used to visualize significant DEGs, confirm the different expression profiles and proportion of up- and down-regulated DEGs in BC tumor tissues from women (Fig. 2A) and men (Fig. 2B) compared to the corresponding BM tissues. Genes with the highest expression fold change (FC ≤ 0.5 or ≥ 2) that were also statistically significant (*p*_adj_ ≤ 0.05) were visually identified as up- or down-regulated.

The top five DEGs most significantly differentiating between BC and BM tissues include *MT-ND6* (FC = 0.08; *p*_adj_ = 6.52E-07), *ARL4C* (FC = 0.39; *p*_adj_ = 6.01E-06), *ASGR1* (FC = 0.06; *p*_adj_ = 1.21E-05), *MYBL1* (FC = 0.17; *p*_adj_ = 1.61E-05) and *SCAMP5* (FC = 2.84; *p*_adj_ = 2.46E-05) in female samples, and *ONECUT2* (FC = 15.54; *p*_adj_ = 2.63E-11), *SPEG* (FC = 0.23; *p*_adj_ = 7.65E-11), *CTSE* (FC = 20.38; *p*_adj_ = 1.13E-10), *GJB2* (FC = 7.36; *p*_adj_ = 1.13E-10) and *SYNM* (FC = 0.17; *p*_adj_ = 1.58E-10) in men samples (Fig. 2 and Supplementary Tables S1 and S2).

### Venn diagram and functional enrichment analysis

**Fig. 3:**
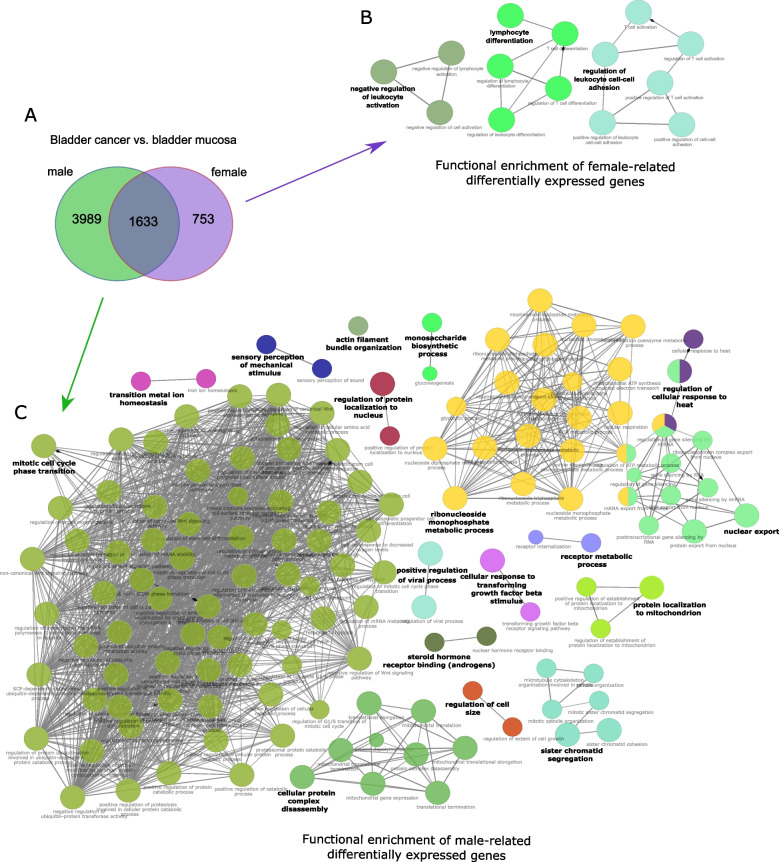
Functional enrichment analysis of unique sex-related differentially expressed genes (DEGs). **A** Venn diagram depict the unique and shared DEGs in bladder cancer and healthy-appearing bladder mucosa tissue comparisons (BC vs. BM) for female and male samples. The top overrepresented pathways identified through ClueGO functional enrichment analysis of unique sex-related DEGs. The BP and MF term categories of the GO database were used to annotate functions to DEGs unique for (**B**) female samples and (**C**) male samples

As shown in the Venn diagram, among the protein-coding DEGs identified in the BC and BM comparisons for each sex, 753 were unique to female samples, 3989 were unique to male samples, and 1633 were common (Fig. 3A). Sets of unique DEGs for both female and male samples were further explore to search for sex-related differences in gene expression profiles, using the BP and MF term categories of the GO database to assign DEG functions. Assuming the involvement of at least 20 genes and 10% of genes in a pathway, ClueGO functional analyses showed that 753 female-unique DEGs significantly (*p*_adj_ < 0.05) enriched 14 pathways that could be further sorted into three GO categories, including *Regulation of leukocyte cell–cell adhesion, Lymphocyte differentiation* and *Negative regulation of leukocyte activation* (Fig. 3B and Supplementary Table S3). In turn, 3989 male-unique DEGs were annotated to 139 pathways that were further divided into 17 categories according to their function (Fig. 3C and Supplementary Table S4). The most enriched GO annotation were related to *Mitotic cell cycle phase transition, Ribonucleoside monophosphate metabolic process, Nuclear export, Cellular protein complex disassembly, Sister chromatid segregation, Protein localization to mitochondrion*, *Regulation of cellular response to heat* and* Steroid hormone receptor binding (androgens).*

**Fig. 4:**
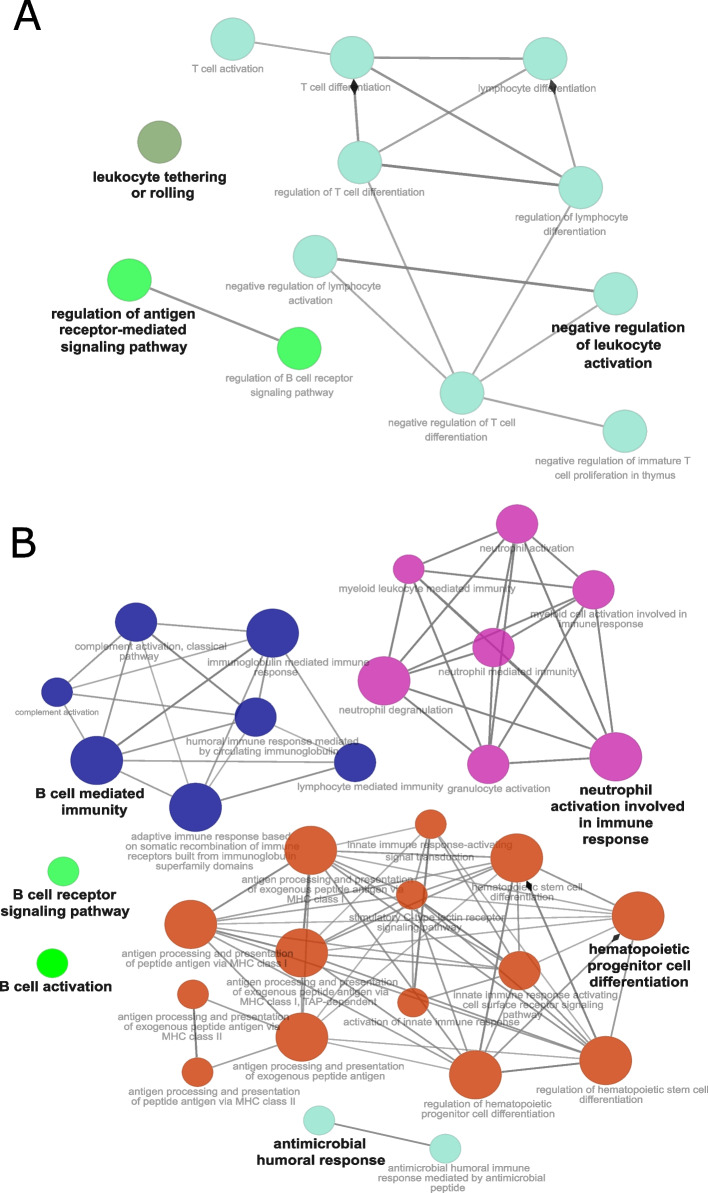
The top overrepresented immune system-related GO pathways identified through functional enrichment analysis of unique sex-related DEGs. GO immune system database was used to annotate functions to DEGs unique for (**A**) female samples and (**B**) male samples

Further, searching for functional annotation to immune system-related GO categories revealed that female-related unique DEGs were annotated to *Negative regulation of leukocyte activation, Regulation of antigen receptor-mediated signaling pathway* and *Leukocyte tethering or rolling* main categories (Fig. 4A and Supplementary Table S5), whereas DEGs unique to men enriched pathways were related to *Hematopoietic progenitor cell differentiation, Neutrophil activation involved in immune response, B cell mediated immunity, Antimicrobial humoral response, B cell receptor signaling pathway* and *B cell activation* (Fig. 4B and Supplementary Table S6).

### The two-factor interaction analysis

Next, to identify those genes whose altered expression in the tumor is gender-related, a two-factor (sex*tissue) model was performed on four transcription data sets, i.e., for BC and BM tissue samples from women and men (men and BM as the baseline and no missing data in the transcription datasets were used for modeling). As shown in Table 2, only *S100A14* protein-coding gene expression in the tumor is gender dependent with significance *p*_adj_-value of less than 0.05. In addition, a probable significant (*p*_adj_ < 0.1) interaction effect was observed for four following protein-coding genes: *GJB2, DSC2, TM4SF1* and *ALOX15B.* The expression of all of these genes in low-grade Ta BC shows a negative interaction effect with female gender, which means that BC vs. adjacent BM tissue expression difference is smaller in female tissues compared with males.

**Table 2:** The two-factor interaction model analysis (Sex_F*tissue_BC)

Gene	FC	*p*_adj_-value	*p*-value
***S100A14***	0.147	**0.0191**	1,9981E-06
*GJB*	0.119	0.0598	1,2938E-05
*DSC2*	0.173	0.0598	1,5678E-05
*TM4SF1*	0.192	0.0676	2,126E-05
*ALOX15B*	0.106	0.0885	3,2447E-05

Bold indicated gene of the Benjamini–Hochberg algorithm adjusted* p*-value (*p*_adj_) ≤ 0.05. *FC* Fold change, *BC* Bladder cancer

Supplementary Table S7 provided all details of two-factor (sex*tissue) analysis including effect size confidence intervals estimated with bootstrap method using 200 iterations. The bootstrap analysis confirms the variability and directionality of the observed effects, enhancing the interpretability of the interaction terms. Furthermore, the boxplots for the five key genes identified in the two-factor interaction model were generated, illustrating sex-dependent expression changes in tumor tissue (Supplementary Figure S2).

## Discussion

The results of our study reveal substantial sex-specific differences in gene expression profiles between BC and adjacent BM tissues, emphasizing the need to integrate biological sex into precision medicine strategies for BC treatment. Furthermore, our analysis identified key DEGs that significantly distinguish BC from BM tissues. In female samples, the most prominent genes include *MT-ND6, ARL4C, ASGR1, MYBL1*, and *SCAMP5*, whereas in male samples, the top differentiating genes are *ONECUT2, SPEG, CTSE, GJB2*, and *SYNM*. Interestingly, most of them have been confirmed to participate in processes related to tumorigenesis and cancer progression, including BC.

*MT‑ND6*, a mitochondrial gene encoding NADH dehydrogenase subunit 6, was markedly downregulated in female BC samples. Mitochondrial dysfunction and reprogramming of oxidative phosphorylation are well-documented contributors to cancer metabolism, including in genitourinary tumors such as BC [17, 18].Alterations in mitochondrial gene expression may influence reactive oxygen species production and apoptotic signaling, processes that are increasingly recognized as modulators of tumor progression [19]. Additionally, mitochondrial gene dysregulation has been linked to therapy resistance and metabolic plasticity in urothelial cancers, suggesting that *MT‑ND6* downregulation could have functional implications beyond energy metabolism [20].

ARL4C, a member of the ADP-ribosylation factor family of GTP-binding proteins, probably involved in microtubule-dependent intracellular vesicular transport, has been associated with tumor progression in various cancers [21–23]. In BC, it enhances metastasis through the activation of the Wnt/β-catenin signaling pathway, as shown by increased levels of cyclin D1 and c-Myc, promoting epithelial-mesenchymal transition [24]. Its role in endometrial and ovarian cancers supports its involvement in tumorigenesis through similar mechanisms [22, 23]. Overexpression of *ARL4C* has also been noted in pancreatic cancer, linking it to poor outcomes [25].

*ASGR1* encodes a subunit of the asialoglycoprotein receptor and mediates the endocytosis of plasma glycoproteins. It has been extensively studied in liver cancer, where it inhibits tyrosine phosphorylation of STAT3 by interacting with Nemo-like kinase (NLK), and plays a significant role in tumor progression [26]. However, the direct role of *ASGR1* in BC remains to be clarified. Similarly MYBL1, a transcription factor, has been widely linked to cancer development [27, 28]. Studies in bladder [27] and breast [28] cancers underscore its role in regulating cell cycle and apoptosis, suggesting its contribution to tumor cell survival. Its influence on BC aligns with its general oncogenic potential, as evidenced by the negative correlation between MYBL1 regulon activity and β-barrel heme protein THAP4, a component of the antioxidant defense system, potentially contributing to malignancy [27]. SCAMP5, involved in vesicle trafficking, ion channel regulation and cytokine exocytosis, has shown oncogenic properties in breast cancer [29], potentially through altered intracellular signaling and metabolic pathways. While less is known about its function in BC, its role in other malignancies indicates it could influence cellular processes relevant to tumor progression [30].

In turn, *ONECUT2*, encoding a member of the onecut family of transcription factors, has been identified as a significant gene in BC. Its methylation is a commonly observed epigenetic alteration, especially in older individuals, suggesting a potential age-related pattern in its regulation and potential usefulness as a predictive biomarker, though its specific role in BC progression remains underexplored [31]. *ONECUT2* has been also linked to neuroendocrine prostate cancer, where it plays a key role in cancer progression and promoting resistance mechanisms, partly by regulating AR signaling [32]. Wang et al. [33] explored sex-specific pathways in BC, revealing that the AR pathway was unique to male hub genes.

Meta-analyze has shown that AR expression correlates with lower tumor grade, earlier stage, and reduced recurrence risk of BC [34]. Decreased AR expression has been linked to more advanced BC stages, with lower levels observed in high-grade and muscle-invasive BC (MIBC) compared to non-invasive cases [34]. Additionally, reduced levels of AR and enzymes involved in androgen metabolism have been associated with BC progression [35]. Emerging evidence suggests that AR may influence tumor differentiation through its interaction with *GATA3* gene, a key regulator of luminal identity in both breast and BC [36]. Kettunen et al. [37] demonstrated that both bladder tumors and non-tumor tissues exhibit extremely low androgen levels, with dihydrotestosterone being nearly undetectable and testosterone remaining at castration levels. Enzymes responsible for androgen inactivation, including HSD17B2, were downregulated in more aggressive tumors, although the expression of androgen-activating enzymes showed little variation. It is suggested that differences in steroid profiles may be linked to tumor aggressiveness, as patients with overall lower circulating steroid levels and those in whom AR expression correlated with intratumorally testosterone levels experienced fewer recurrences [37].

*SPEG*, encoding a striated muscle-enriched protein kinase involved in cytoskeletal organization and intracellular signaling [38], showed reduced expression in male BC samples. It is integrated into gene signatures associated with focal adhesion and cytoskeletal remodeling [39]. Given that kinases of this family are pivotal in regulating cell adhesion, migration, and contractility, SPEG may similarly contribute to the invasive phenotype of BC cells [40], however, direct evidence linking *SPEG* to BC biology remains limited.

Cathepsin E encoded by *CTSE*, has shown a male-biased expression pattern in BC, with a male-to-female ratio of 3.64 [41]. CTSE could serve as an independent prognostic marker for NMIBC, potentially aiding in treatment decisions [41]. Blaveri et al. [42] observed higher *CTSE* expression in NIMBC compared to MIBC. Additionally, a long-term follow-up study of NMIBC cases confirmed that lower *CTSE* expression was significantly correlated with the progression from NMIBC to MIBC [43]. *CTSE* has been also identified as being differentially expressed in pancreatic tumors, with its expression levels closely linked to tumor initiation and progression [44]. In turn, SYNM, a member of an intermediate filament family of cytoskeletal proteins, was associated with tumor invasiveness and may influence cell adhesion and migration, crucial for metastasis development and tumor progression [45]. In a survival analysis conducted by Wang et al. [46], several hub genes, including *SYNM*, *CNKSR1* and *POPDC2* were identified as being associated with the progression and prognosis of patients with MIBC. The expression of *SYNM* in BC may indicate its role in facilitating cancer cell adaptation, allowing cells to better survive and proliferate under challenging conditions, such as hypoxia or oxidative stress. In turn, *GJB2*, encoding connexin 26, has been implicated in BC progression, with its overexpression promoting tumor cell proliferation and invasion [47].

The two-factor interaction analysis identified *S100A14* as a protein-coding gene with a significant gender-dependent expression in low-grade Ta BC. Additionally, probable significant interaction effects were observed for *GJB2*, *DSC2*, *TM4SF1*, and *ALOX15B*, all showing a negative interaction with female gender. However, caution is warranted as these interaction effects did not reach conventional significance thresholds and should be regarded exploratory signals requiring validation in larger cohorts. The *S100A14* gene encoded a calcium-binding protein, whose role in cellular signaling pathways is only partially understood. S100A14 played a role in various cancer-associated processes, including cellular differentiation, invasion or migration [48]. Its expression patterns and subcellular localization varied significantly across cancers, which explained its specific roles in different tumor types [48–50]. S100A14 was proposed to participate in diverse signaling cascades, with outcomes varying greatly depending on the tumor type and context. Similar to other S100 family members, S100A14 interacted with transmembrane receptors such as HER2/ErbB2 and RAGE.

Katono et al. [51] demonstrated that *S100A14* expression in lung adenocarcinoma was significantly associated with sex; potentially due to its relationship with oncogenic driver mutations, including alterations in *ALK* or *EGFR* [52]. This observation highlights the potential role of S100A14 in sex-biased tumor biology, particularly in epithelial cancers. In lung adenocarcinoma, *S100A14* expression has been also linked to differential signaling pathways that may be modulated by hormonal factors. It interacts with key signaling molecules such as MMPs, RAGE, HER2, and NF-κB, and modulates cytoskeletal dynamics and immune signaling [53, 54]. These pathways are known to differ between sexes and may contribute to sex-specific patterns of tumor progression. In breast cancer, co-expression of *S100A14* and *S100A16* correlates with poor prognosis and increased invasiveness, particularly in ER-negative and HER2-positive tumors [54, 55]. Although direct evidence in BC is limited, the parallels in epithelial origin and immune involvement support the hypothesis that S100A14 may play a similar sex-modulated role in bladder tumorigenesis [53]. Additionally, S100A14 showed a close functional association with the tumor suppressor p53 in cancer biology. S100A14 demonstrated potential as a therapeutic target in various diseases, including BC, due to its role in regulating tumor progression and metastasis [49, 50]. Its dual function as both a tumor suppressor and promoter, depending on the context, underscored its importance in cancer research and treatment approaches. However, this is the first time that its gender-related contribution to low-grade Ta BC has been indicated.

Beyond its role in promoting proliferation and invasion, GJB2 has also been implicated in modulating the tumor immune microenvironment. Pan-cancer analyses revealed associations between *GJB2* expression and immune checkpoint gene regulation, tumor mutational burden, and immune cell infiltration [56]. In lung adenocarcinoma, GJB2 enhances cancer stem cell properties via NF-κB and SOX2 activation [57]. DSC2 is a desmosomal cadherin involved in epithelial adhesion and immune cell interaction. In osteosarcoma, high *DSC2* expression correlates with poor prognosis and immune infiltration [58]. In breast cancer, *DSC2* depletion promotes proliferation and migration via AKT and ERK signaling, with distinct mechanisms in luminal vs. triple-negative subtypes [59]. Although sex-specific expression is not well documented, its role in epithelial cohesion and immune modulation suggests relevance to sex-influenced tumor biology.

Li et al. [60] demonstrated that *ALOX15B* was significantly downregulated in human BC cell lines. Knockdown of *ALOX15B* enhanced BC cell growth and protected the cells from p53-induced ferroptosis. Additionally, p53 was found to activate *ALOX15B* lipoxygenase activity by inhibiting *SLC7A11*, thereby promoting ferroptosis in BC cells. ALOX15B is a non-heme iron dioxygenase involved in lipid metabolism and immune regulation. It promotes ferroptosis and inhibits proliferation in bladder and breast cancer cells [61, 62]. Its expression is induced by hypoxia and linked to cholesterol homeostasis in macrophages, suggesting a role in immune cell function and epithelial remodeling [61]. In breast cancer, ALOX15B-based efferocytosis clusters are associated with favorable prognosis and immune infiltration, particularly in luminal subtypes [62].

As demonstrated by the study of Cao et al. [63], *TM4SF1*, a member of the L6 transmembrane protein family, was highly overexpressed in MIBC tissues. Its increased expression correlated with advanced tumor stage, lymph node metastasis, and poorer patient prognosis. Functional experiments revealed that silencing of *TM4SF1* suppressed BC cell proliferation in vitro and in vivo, leading to cell cycle arrest and apoptosis, likely due to elevated levels of reactive oxygen species. Furthermore, blocking PPARγ or activating *SIRT1* reversed these effects [64]. Similarly, the study by Yang et al. [65] indicated that *TM4SF1* is overexpressed in histologic variants of BC, which are more aggressive and resistant to therapy. Both these studies highlights its potential as a therapeutic target in BC [64]. TM4SF1 promotes epithelial-mesenchymal transition, invadopodia formation, and immune evasion [66]. Although sex-specific expression has not been confirmed, TM4SF1 is involved in epithelial and immune interactions that may be modulated by sex-dependent factors.

In our study, functional enrichment analysis revealed that female-specific DEGs were predominantly associated with processes related to regulation of leukocyte cell–cell adhesion and activation and lymphocyte differentiation*.* In contrast, male-specific DEGs were predominantly enriched in pathways associated with the regulation of the mitotic cell cycle, and cell proliferation in response to heat and hormonal signals. Similarly to our findings, Wang et al. [67], found that DEGs between benign mucosa and control groups were primarily involved in cell cycle processes and chromosomal assembly, including spindle checkpoint regulation, mitotic sister chromatid segregation, and DNA replication. However, the authors did not stratify the BC cases based on sex. In turn, Tang et al. [68] demonstrated that upregulated DEGs in BC samples compared to non-cancerous samples were primarily associated with mitotic cell cycle and division, and protein binding. Considering that the study involved almost exclusively men (39 vs. 4 females), the result seems also consistent with ours.

Upon further functional annotation, male-specific genes were also linked to immune system-related GO terms, including B cell-driven humoral immune response. Bulk RNA sequencing and multiplex immunofluorescence demonstrated extensive immune landscape remodeling with aging, particularly in B cell-mediated responses in healthy bladder tissue [69]. Analysis showed enrichment of B cell receptor signaling and humoral immunity pathways starting at 12 months, with 18-month-old females exhibiting a significantly higher enrichment score than males. Functional enrichment analysis further confirmed that age-related clustering of B cell-associated pathways occurred in both sexes, but the extent of these changes was more pronounced in females [69].

Chenard et al. [12] described variations in immune profiles of BC tumors based on sex. Comparing with low-grade tumors in both sexes, high-grade tumors in females exhibited significantly higher levels of immune checkpoint genes, including *LAG3, ICOS, PDCD1*, and *CTLA4*, than those in males. Additionally, the expression of the B-cell-recruiting chemokine *CXCL13* and the surface molecule *CD40* was elevated. Increased infiltration of CD163 + tumor-associated macrophages was found in the epithelial and stromal areas of high-grade tumors in women, as well as in the epithelial areas of low-grade tumors, compared to men. Moreover, the epithelial compartments of low-grade tumors in women showed significantly higher PD-L1 protein expression compared to those in men. We have shown that in both sexes, DEGs enrich pathways related to immune mechanisms; however, these pathways differ between sexes, with sex-specific variations in immune regulation, cell adhesion, and cell cycle processes potentially influencing susceptibility to BC and its progression.

Although our study does not include clinical follow-up data, the observed sex-specific transcriptomic differences may have implications for prognosis and therapeutic decision-making in BC. For instance, the enrichment of immune-related pathways in female patients could influence responsiveness to immunotherapies, while the predominance of cell cycle and androgen-related pathways in males may suggest differential sensitivity to targeted treatments. Understanding these molecular distinctions could eventually support the development of sex-informed biomarkers and personalized therapeutic strategies aimed at improving outcomes and reducing recurrence rates in both sexes.

### Limitations and methodological considerations

Our study has several limitations that warrant careful consideration. First, the relatively small cohort size, although strengthened by the paired design, limits the statistical power, particularly for detecting subtle interaction effects such as sex*tumor status. Bootstrap confidence intervals and FDR adjustments were applied to enhance robustness, however, these approaches cannot fully compensate for the limited sample size and should not be interpreted as definitive evidence. While the applied statistical thresholds and multiple testing corrections reduce the risk of false positives, the limited sample size may have led to under-detection of DEGs, especially those with moderate effect sizes. Additionally, the interaction analysis identified only a few genes with statistically significant sex-dependent expression patterns, with *S100A14* being the sole gene meeting the *p*_adj_ < 0.05 threshold. This limited signal likely reflects the constrained sample size and reduced statistical power for detecting interaction effects. Therefore, the interaction findings should be interpretedwith caution and considered hypothesis-generating rather than confirmatory.

Second, the comparison between tumor tissue and adjacent healthy mucosa introduces potential confounding due to differences in cellular composition, including immune and stromal cell infiltration. This is particularly relevant for the observed enrichment of immune-related pathways among female-specific DEGs, which could partly reflect microenvironmental rather than tumor-intrinsic changes. Although the paired sampling strategy helps control for inter-individual variability, bulk RNA-seq does not allow for precise deconvolution of cell-type-specific expression. Additionally, the possibility of field cancerization, molecular alterations in histologically normal adjacent mucosa, cannot be excluded and may differ between sexes. Field cancerization refers to the presence of genetically or epigenetically altered cells in tissue that appears histologically normal, often due to chronic exposure to carcinogens or tumor-derived signaling [70]. Such alterations may be influenced by sex-specific factors, including hormonal regulation, immune responses, and differential susceptibility to mutagenic processes [71]. Future studies with larger cohorts and complementary validation approaches, such as single-cell or spatial transcriptomics, will be essential to confirm the sex-dependent expression patterns suggested by our data and to distinguish tumor-intrinsic gene expression from microenvironmental influences. In this context, we acknowledge that the use of adjacent mucosa as a control tissue may introduce bias, particularly in studies of sex-dependent gene expression, due to potential field cancerization and sex-specific microenvironmental effects [72]. Future research should therefore consider distant normal tissue or sex-stratified sampling strategies to mitigate this limitation and improve interpretability.

In addition to the methodological considerations discussed above, we further acknowledge limitations that may impact the generalizability and translational potential of our findings. Due to the markedly higher incidence of BC in men and the complex nature of tumor biology, constructing well-balanced patient groups with respect to sex and disease characteristics remains challenging. Despite the relatively small sample size, our study was sufficiently powered to detect significant sex-related differences in low-grade and non-invasive tumors, which may serve as a foundation for future functional studies. Importantly, the current analysis is based solely on WTS, and lacks complementary proteomic and metabolomic validation. Integrating multi-omics approaches in future research would provide a more comprehensive understanding of sex-specific molecular mechanisms in BC and enhance the clinical relevance of our observations.

Overall, while our findings highlight intriguing sex-related transcriptomic differences, they should be interpreted with caution given the limited statistical power and potential confounding by adjacent tissue effects. These results are exploratory and hypothesis-generating rather than confirmatory. Future studies employing single-cell or spatial transcriptomics and larger, sex-balanced cohorts will be essential to validate these preliminary observations and clarify the biological mechanisms underlying sex-specific differences in BC.

## Conclusion

Our findings suggest that sex-specific molecular mechanisms may contribute to BC biology and progression. Considering the distinct transcriptomic patterns observed in male and female patients, future research should focus on elucidating the precise roles of these DEGs and their potential relevance to sex-dependent therapeutic strategies. These preliminary findings warrant validation in larger cohorts and functional studies to determine whether targeting sex-related differences could improve clinical outcomes and reduce recurrence rates.

## Supplementary Information

Supplementary Material 1: Supplementary Figure S1. The proportion of mitochondrial reads per sample calculated by ANOVA. Supplementary Figure S2. The boxplots for the five key genes identified in the two-factor interaction model illustrating sex-dependent expression changes in tumor tissue. Supplementary Table S1. DEGs most significantly differentiating between BC (g) and BM (s) tissues in female samples. Supplementary Table S2. DEGs most significantly differentiating between BC (g) and BM (s) tissues in male samples. Supplementary Table S3. Significantly enriched GO categories in ClueGO functional analysis of 753 female-unique DEGs (*p*_adj_ < 0.05). Supplementary Table S4. Significantly enriched GO categories in ClueGO functional analysis of 3989 male-unique DEGs (*p*_adj_ < 0.05). Supplementary Table S5. Functional annotation to immune system-related GO categories of female-related unique DEGs. Supplementary Table S6. Functional annotation to immune system-related GO categories of male-related unique DEGs. Supplementary Table S7. Two-factor (sex*tissue) interaction model. Sex-related gene altered expression in bladder tumor samples; Tissue: bladder cancer (BC), bladder mucosa (BM); Sex: female (F), male (M).

## Data Availability

The data that support the findings of this study were deposited at GEO repository under accession number GSE293398.

## Abbreviations

AR: Androgen receptor
BC: Bladder cancer
BM: Bladder mucosa
BP: Biological processes
DEG: Differentially expressed gene
FC: Fold change
GO: Gene ontology
MF: Molecular function
MIBC: Muscle-invasive bladder cancer
NMIBC: Non-muscle-invasive bladder cancers
PCA: Principal component analysis
TURBT: Transurethral resection of the bladder tumor
WTS: Whole transcriptome sequencing
