# Supplementary figures and images for: Sex-related differences in gene expression in early-stage bladder cancer revealed by whole-transcriptome sequencing
Source: BMC Cancer. 2026 Feb 9;26:361. doi: 10.1186/s12885-026-15666-3 (PMC12998147; doi:10.1186/s12885-026-15666-3)

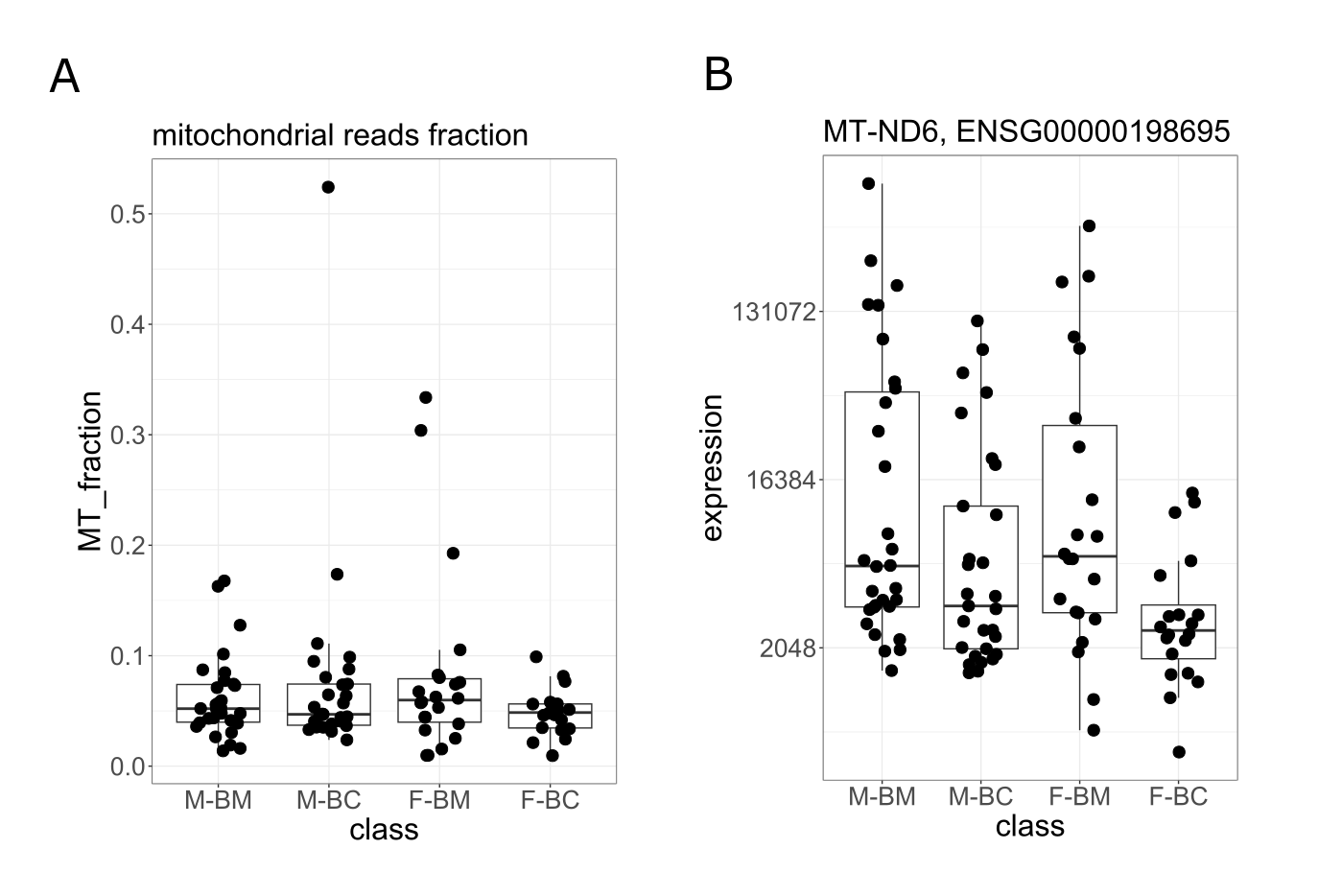

Supplement: Supplementary file 1 — Supplementary Material 1: Supplementary Figure S1. The proportion of mitochondrial reads per sample calculated by ANOVA. Supplementary Figure S2. The boxplots for the five key genes identified in the two-factor interaction model illustrating sex-dependent expression changes in tumor tissue. Supplementary Table S1. DEGs most significantly differentiating between BC (g) and BM (s) tissues in female samples. Supplementary Table S2. DEGs most significantly differentiating between BC (g) and BM (s) tissues in male samples. Supplementary Table S3. Significantly enriched GO categories in ClueGO functional analysis of 753 female-unique DEGs (padj < 0.05). Supplementary Table S4. Significantly enriched GO categories in ClueGO functional analysis of 3989 male-unique DEGs (padj < 0.05). Supplementary Table S5. Functional annotation to immune system-related GO categories of female-related unique DEGs. Supplementary Table S6. Functional annotation to immune system-related GO categories of male-related unique DEGs. Supplementary Table S7. Two-factor (sex*tissue) interaction model. Sex-related gene altered expression in bladder tumor samples; Tissue: bladder cancer (BC), bladder mucosa (BM); Sex: female (F), male (M). [file 12885_2026_15666_MOESM1_ESM.zip › Supplementary Figure S1.png]

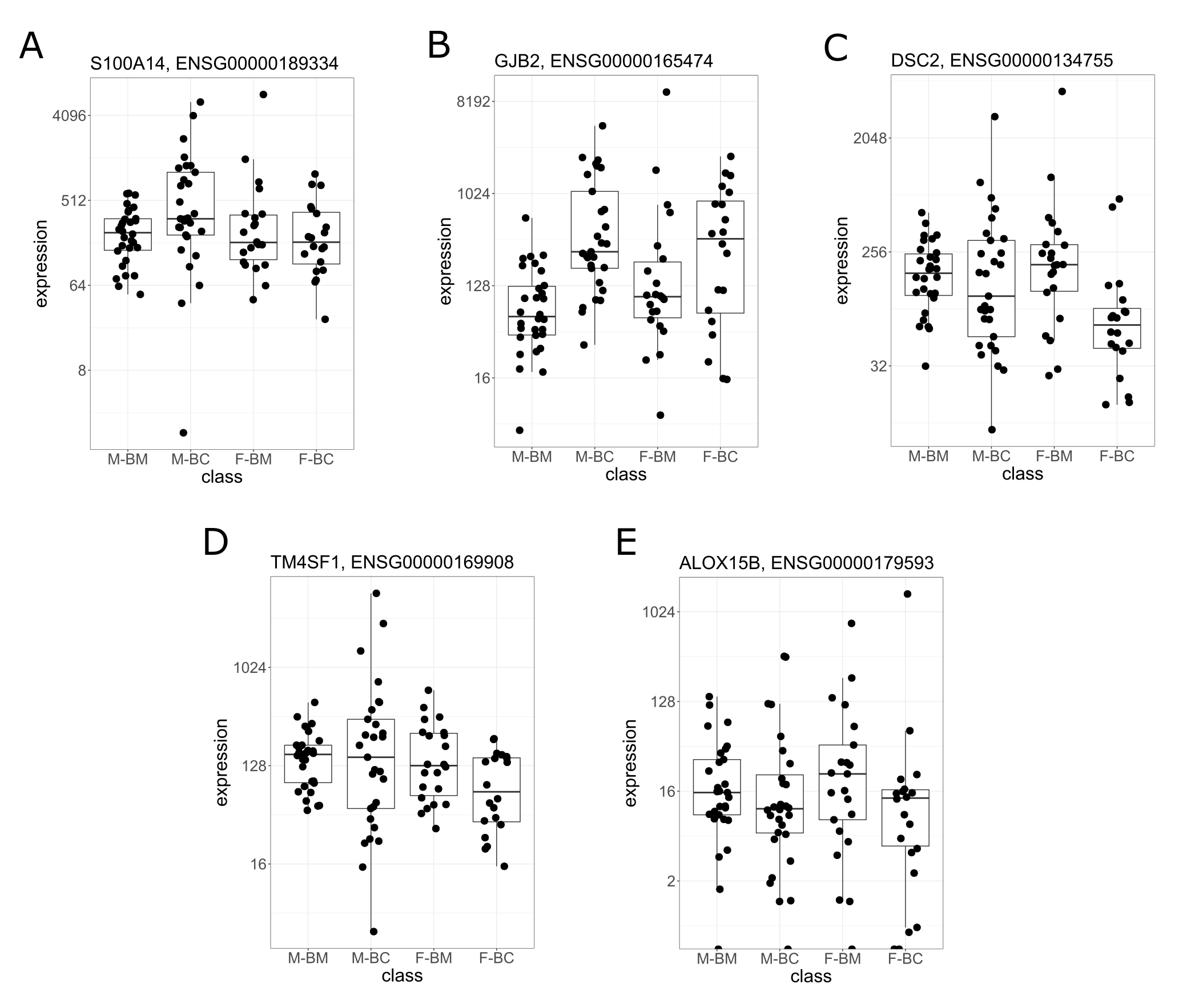

Supplement: Supplementary file 1 — Supplementary Material 1: Supplementary Figure S1. The proportion of mitochondrial reads per sample calculated by ANOVA. Supplementary Figure S2. The boxplots for the five key genes identified in the two-factor interaction model illustrating sex-dependent expression changes in tumor tissue. Supplementary Table S1. DEGs most significantly differentiating between BC (g) and BM (s) tissues in female samples. Supplementary Table S2. DEGs most significantly differentiating between BC (g) and BM (s) tissues in male samples. Supplementary Table S3. Significantly enriched GO categories in ClueGO functional analysis of 753 female-unique DEGs (padj < 0.05). Supplementary Table S4. Significantly enriched GO categories in ClueGO functional analysis of 3989 male-unique DEGs (padj < 0.05). Supplementary Table S5. Functional annotation to immune system-related GO categories of female-related unique DEGs. Supplementary Table S6. Functional annotation to immune system-related GO categories of male-related unique DEGs. Supplementary Table S7. Two-factor (sex*tissue) interaction model. Sex-related gene altered expression in bladder tumor samples; Tissue: bladder cancer (BC), bladder mucosa (BM); Sex: female (F), male (M). [file 12885_2026_15666_MOESM1_ESM.zip › Supplementary Figure S2.png]
